# High clinical diagnostic accuracy of combined salivary gland and myocardial metaiodobenzylguanidine scintigraphy in the diagnosis of Parkinson’s disease

**DOI:** 10.3389/fnagi.2022.1066331

**Published:** 2023-01-11

**Authors:** Shuangfang Li, Lei Yue, Shuzhen Chen, Zhuang Wu, Jingxing Zhang, Ronghua Hong, Ludi Xie, Kangwen Peng, Chenghong Wang, Ao Lin, Lingjing Jin, Qiang Guan

**Affiliations:** ^1^Neurotoxin Research Center of Key Laboratory of Spine and Spinal Cord Injury Repair and Regeneration of Ministry of Education, Department of Neurology, Tongji Hospital, School of Medicine, Tongji University, Shanghai, China; ^2^Department of Neurology and Neurological Rehabilitation, Shanghai YangZhi Rehabilitation Hospital (Shanghai Sunshine Rehabilitation Center), School of Medicine, Tongji University, Shanghai, China; ^3^Department of Nuclear Medicine, Tongji Hospital, School of Medicine, Tongji University, Shanghai, China; ^4^Shanghai Clinical Research Center for Aging and Medicine, Shanghai, China

**Keywords:** Parkinson’s disease, parkinsonism, salivary glands, MIBG scintigraphy, diagnosis

## Abstract

**Background:**

Decreased myocardial uptake of ^131^I-metaiodobenzylguanidine (MIBG) is known to be an important feature to diagnose Parkinson’s disease (PD). However, the diagnosis accuracy of myocardial MIBG scintigraphy alone is often unsatisfying. Recent studies have found that the MIBG uptake of the major salivary glands was reduced in PD patients as well.

**Purpose:**

To evaluate the diagnostic value of major salivary gland MIBG scintigraphy in PD, and explore the potential role of myocardial MIBG scintigraphy combined with salivary gland MIBG scintigraphy in distinguishing PD from non-PD (NPD).

**Methods:**

Thirty-seven subjects were performed with ^131^I-MIBG scintigraphy. They were classified into the PD group (*N* = 18) and the NPD group (*N* = 19), based on clinical diagnostic criteria, DAT PET and ^18^F-FDG PET imaging findings. Images of salivary glands and myocardium were outlined to calculated the MIBG uptake ratios.

**Results:**

The combination of left parotid and left submandibular gland early images had a good performance in distinguishing PD from NPD, with sensitivity, specificity, and accuracy of 50.00, 94.74, and 72.37%, respectively. Combining the major salivary gland and myocardial scintigraphy results in the early period showed a good diagnostic value with AUC, sensitivity and specificity of 0.877, 77.78, and 94.74%, respectively. Meanwhile, in the delayed period yield an excellent diagnostic value with AUC, sensitivity and specificity of 0.904, 88.89, and 84.21%, respectively.

**Conclusion:**

^131^I-MIBG salivary gland scintigraphy assisted in the diagnosis and differential diagnosis of PD. The combination of major salivary gland and myocardial ^131^I-MIBG scintigraphy further increased the accuracy of PD diagnosis.

## 1. Introduction

Parkinson’s disease (PD) is a common progressive neurological illness with insidious onset and slow progression (De Rijk et al., 1997). As no biomarkers are available, the diagnosis of PD is mainly based on clinical criteria but the accuracy is limited, especially in the early stages because of lacking typical clinical signs and symptoms (Adler et al., 2014). Similar motor symptoms, such as resting tremor or bradykinesia, occur in atypical parkinsonian syndromes (APS), vascular parkinsonism (VaP) and essential tremor (ET) (Lees et al., 2009). However, the prognosis of these diseases is completely different, and therefore, improving diagnostic accuracy is very important for the choice of a therapeutic approach.

PD is characterized by abnormal deposition of α-synuclein aggregates forming intraneuronal Lewy bodies and Lewy neurites, which are found in many regions of the central nervous system and peripheral tissues, such as the heart, salivary glands and gut (Den Hartog Jager and Bethlem, 1960; Beach et al., 2010; Del Tredici et al., 2010; Tsukita et al., 2019). Degeneration of the cardiac sympathetic system due to abnormal aggregation of α-synuclein has been used to diagnose PD by means of metaiodobenzylguanidine (MIBG) scintigraphy (Orimo et al., 2008). The new clinical diagnostic criteria for PD proposed by the International Parkinson and Movement Disorder Society (MDS) in 2015 used cardiac sympathetic denervation on MIBG scintigraphy as a supportive criterion (Postuma et al., 2015). Many studies have reported that myocardial MIBG uptake decreases in PD patients with disease progression (Spiegel et al., 2005; Ryu et al., 2019), and myocardial MIBG scintigraphy is significant in the differential diagnosis of PD and other forms of parkinsonism, such as multiple system atrophy (MSA), progressive supranuclear palsy (PSP), corticobasal degeneration (CBD), vascular parkinsonism, etc., and in the discrimination from healthy people (Satoh et al., 1999; Orimo et al., 2012; Treglia et al., 2012). However, the diagnostic value of this test can be affected when PD patients experience concurrent damage to the cardiac muscle, such as ischemic heart disease, myocardiopathy, and congestive heart failure (Braune, 2001; Jacobson et al., 2010).

Recent studies have found that the sympathetic nerves to the salivary glands are also damaged, and MIBG scintigraphy of the major salivary glands has shown reduced uptake in PD patients (Haqparwar et al., 2017; Campo et al., 2019; Schepici et al., 2020). Normal MIBG uptake in healthy people and patients with PSP, MSA, and CBD is consistent with myocardial MIBG scintigraphy (Haqparwar et al., 2017; Schubert et al., 2019). Therefore, we speculate that salivary gland MIBG scintigraphy may have some clinical value and can improve the diagnostic value of myocardial MIBG scintigraphy when used in combination in PD patients. In previous studies, planar whole-body images were mostly collected, and the regions of interest (ROIs) in the relevant parts were delineated to calculate the radioactive uptake ratio. Concomitantly, in planar images, lung uptake or liver uptake superimposed on cardiac uptake may cause false-negative and false-positive results (Oh et al., 2015). Tomographic images are superior imaging techniques for the evaluation of specific organ activity (Fukuoka et al., 2011). In our study, single photon emission computed tomography (SPECT/CT) images were used to further evaluate the salivary glands and cardiac sympathetic denervation.

Accordingly, the present study aims to (1) evaluate the diagnostic value of major salivary gland ^131^I-MIBG scintigraphy in PD patients; and (2) explore the potential role of ^131^I-MIBG myocardial scintigraphy in conjunction with ^131^I-MIBG salivary glands scintigraphy in distinguishing PD patients from non-PD (NPD) patients.

## 2. Materials and methods

### 2.1. Participants

The study involved 31 patients who visited the outpatient clinic of the Neurological Department of Tongji Hospital Affiliated to Tongji University from April 2017 to October 2019. The patients chiefly complained of one or more parkinsonian symptoms, including bradykinesia, rigidity or resting tremor. All patients were subjected to a comprehensive clinical evaluation and examination conducted by specialists in movement disorders, and were followed up over a course of 3 to 4 years. Those who met the MDS clinical diagnostic criteria for Parkinson’s disease were diagnosed with clinically definite PD (Postuma et al., 2015), and the myocardial MIBG scintigraphy results were not used as a supportive diagnostic criterion. Others with clinically relevant symptoms of parkinsonian syndrome, but without a diagnosis of clinically definite and possible PD or who did not match the dopamine transporter (DAT) and ^18^F-fluorodeoxyglucose (^18^F-FDG) positron emission tomography (PET) imaging findings of PD patients were classified as NPD by 2 independent neurologists. Referring to a similar diagnostic flow in previous studies (Ishibashi et al., 2010; Stathaki et al., 2020), patients with unclassified Parkinsonian syndrome were included in the NPD group after excluding PD diagnosis. Moreover, considering that this study is an exploration of diagnostic methods, only comparing PD patients with other parkinsonian syndrome or healthy control group will reduce the accuracy of diagnostic tests (Sackett and Haynes, 2002; Rutjes et al., 2006). Therefore, the control group of this study includes patients with Parkinsonian syndrome whose symptoms are easily confused with Parkinson’s disease clinically and some healthy people. We recruited 6 healthy subjects into the NPD group. Motor symptoms were evaluated using the MDS sponsored revision of the Unified Parkinson’s Disease Rating Scale part III (MDS-UPDRS III) (Goetz et al., 2008), and the Hoehn and Yahr (H&Y) scale.

The exclusion criteria for the study included an individual history of salivary glands disease, heart disease, diabetes, hypothyroidism or peripheral neuropathy. None of the patients were on specific medications that would interfere with MIBG uptake, such as calcium antagonists, tricyclic antidepressant medications, labetalol, and ephedrine hydrochloride. ^131^I-MIBG scintigraphy was performed for all subjects.

The Ethics Committee of Tongji Hospital approved the present research (IRB 2018-LCYJ-009-XZ-181105) and the registration number is ChiCTR1800015757. All participants signed written informed consent before the research. All the research procedures were performed according to Helsinki declaration.

### 2.2. ^131^I-MIBG scintigraphy

To block the thyroid gland, all subjects were administered compound iodine solution starting 3 days prior to imaging. On the day of imaging, an intravenous injection of ^131^I-MIBG with a radioactivity of 111 MBq was given. SPECT/CT images of the major salivary glands and the heart were obtained at 30 min for the early images and at 4 h for the delayed images after the injection on a dual-head gamma camera and multidetector (16-row) spiral CT (Precedence SPECT/CT; Philips Healthcare, Amsterdam, Netherlands). The spiral CT examination from head to thorax was performed with the parameters of 100 mAs, 120 keV, and 5-mm section width. SPECT followed a CT scan from head to thorax with a 15% energy window centred on a 364-keV photopeak. Acquisition parameters for SPECT included a 64 × 64 matrix with 64 frames (15 s/frame) over 360°. SPECT data were reconstructed using Astonish methods incorporating photon attenuation correction based on the X-ray transmission map and scatter correction through AutoSPECT+ software. Then the reconstructed SPECT data and CT data were fused and analysed on an EBW workstation, providing the transverse, sagittal, and coronal slices of SPECT, CT, and fused SPECT/CT data.

### 2.3. Imaging analysis

**Figure 1:**
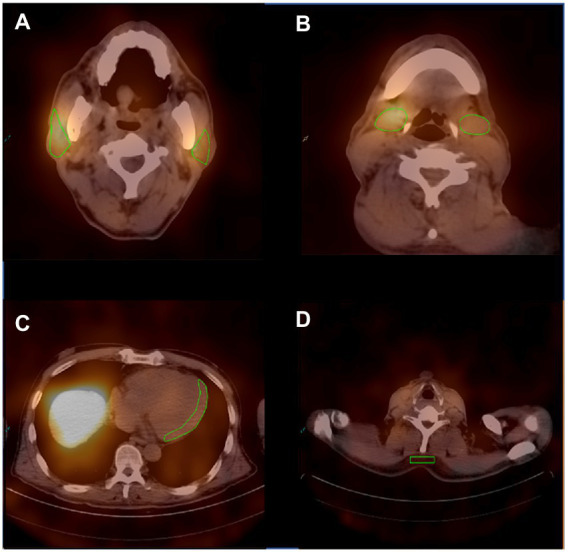
Representative ROIs of MIBG scintigraphy. **(A)** The ROIs of the bilateral parotid glands. **(B)** The ROIs of the bilateral submandibular glands. **(C)** The ROI of the left ventricular wall. **(D)** The ROI of the neck subcutaneous tissue.

For quantification of the relative organ uptake of ^131^I-MIBG, the left ventricular wall, parotid glands and submandibular glandswere contoured manually three times on the fusion images of transverse slices using an ROI technique(Figure 1). A rectangular ROI of the neck subcutaneous tissueand served as the background activity. The mean radioactive values of the left ventricular wall, parotid glands, submandibular glands and background activity were calculated. Care was taken to exclude blood in the ventricle. The radioactive value ratio of the heart to the neck subcutaneous tissue(H/N), and the ratio of the major salivary glands to the neck subcutaneous tissue(P/N or S/N) in the early and delayed periods were calculated. Regarding the P/N and S/N indices, values for the salivary glands on the left and right were used for the analysis.

### 2.4. Statistical analysis

The statistical analyses were performed using SPSS software (IBM SPSS Statistics, version 20, IBM, Corp., Armonk, New York, United States). The normality of the data distribution was estimated by the Shapiro–Wilk test. Differences in the quantitative indices between PD and NPD groups were compared using the Mann–Whitney *U* test or independent sample *t* test. *p* values of <0.05 (two-sided) were considered statistically significant. Figures were produced by GraphPad Prism software (GraphPad Prism Version 8.0, GraphPad software, San Diego, CA, United States).

## 3. Results

According to the clinical diagnosis and evaluation, 18 patients (12 men and 6 women; age range: 42–76 years, mean age ± SD: 57.61 ± 11.09 years) were diagnosed with PD, and 19 patients (7 men and 12 women; age range: 47–78 years, mean age ± SD: 63.95 ± 8.61 years) were classified into NPD group, including 8 MSA patients, 1 PSP patient, 1 VaP patient, 6 healthy individuals and 3 patients for whom the diagnosis could not be defined despite follow-up for at least 3 years. The MDS-UPDRS III score was 42.61 ± 12.20 points (mean ± SD) in PD patients. The distributions of age and sex did not differ between the two groups. Characters and the MIBG uptake ratios of all subjects are given in Supplementary Tables 1, 2.

The salivary gland and myocardial MIBG uptake ratios in the two groups are showed in Table 1. Both in the early and delayed images, the MIBG uptake in the bilateral parotid glands and submandibular glands in the PD group was obviously lower than that in the NPD group. In the early images, the left parotid gland, the left and right submandibular gland MIBG uptake ratios in the PD patients were significantly lower (L-P/N, *p* < 0.05; L-S/N, *p* < 0.05; R-S/N, *p* < 0.05). The MIBG uptake ratio for the left submandibular gland was also significantly decreased in the delayed images (L-S/N, *p* < 0.05). Regarding myocardial MIBG scintigraphy, the differences were more pronounced between the two groups, both in the early and delayed periods, compared to salivary glands images.

**Table 1:** Differences of salivary gland and myocardial MIBG scintigraphy results in PD and NPD.

	PD	NPD	*P* value
Early P/N			
L-P/N	4.74 ± 2.38	6.01 ± 1.88	**0.014** ^*^
R-P/N	5.57 ± 2.28	6.70 ± 1.81	0.103
Early S/N			
L-S/N	4.84 ± 1.76	6.18 ± 1.76	**0.022** ^*^
R-S/N	5.24 ± 2.04	6.27 ± 1.54	**0.034** ^*^
Early H/N	3.37 ± 1.46	6.01 ± 1.80	**<0.001** ^***^
Delayed P/N			
L-P/N	6.01 ± 2.06	7.18 ± 2.51	0.118
R-P/N	6.64 ± 2.00	7.77 ± 2.75	0.258
Delayed S/N			
L-S/N	5.36 ± 2.04	7.04 ± 2.54	**0.034** ^*^
R-S/N	5.69 ± 1.90	6.71 ± 1.69	0.070
Delayed H/N	2.89 ± 2.33	5.22 ± 1.76	**<0.001** ^***^

Bold font means significant results; Data are means ± SD; PD, Parkinson’s disease; NPD, other Parkinsonian syndrome and healthy control; L, left side; R, right side; P/N, the ratio of the parotid glands to the neck subcutaneous tissue; S/N, the ratio of the submandibular glands to the neck subcutaneous tissue; H/N, the ratio of the heart to the neck subcutaneous tissue. ^*^P < 0.05, ^***^P< 0.001.

Table 2 shows the results of the ROC curve analysis as an indicator of the diagnostic abilies of salivary gland and myocardial MIBG values. The OCVs were L-P/N: 4.63, L-S/N: 4.47, R-S/N:6.47, and H/N:3.49 in the early images and L-S/N: 6.99, and H/N: 3.57 in the delayed images.

**Table 2:** Measures of diagnostic accuracy of salivary gland and myocardial MIBG scintigraphy in differentiating PD and NPD.

	AUC	OCV	95% CI	Sensitivity (%)	Specificity (%)	Accuracy (%)
Early L-P/N	**0.734**	4.63	0.567–0.900	66.67	76.68	71.68
Early R-P/N	0.678	5.62	0.502–0.855	66.67	68.42	67.55
Early L-S/N	**0.719**	4.47	0.552–0.886	55.56	84.21	69.89
Early R-S/N	**0.703**	6.47	0.528–0.878	83.33	57.89	70.61
Early H/N	**0.868**	3.49	0.746–0.991	72.22	94.74	83.48
Delayed L-P/N	0.652	4.98	0.471–0.834	55.56	84.21	69.89
Delayed R-P/N	0.610	8.33	0.425–0.794	88.89	36.84	62.87
Delayed L-S/N	**0.703**	6.99	0.532–0.875	88.89	52.63	70.76
Delayed R-S/N	0.674	5.90	0.495–0.853	66.67	68.42	67.55
Delayed H/N	**0.857**	3.57	0.719–0.995	88.89	84.21	86.55

Bold font means significant results; AUC, area under the receiver operating characteristic curve; OCV, the optimal cutoff values; CI, the confidence interval; L, left side; R, right side; P/N, the ratio of the parotid glands to the neck subcutaneous tissue; S/N, the ratio of the submandibular glands to the neck subcutaneous tissue; H/N, the ratio of the heart to the neck subcutaneous tissue.

**Figure 2:**
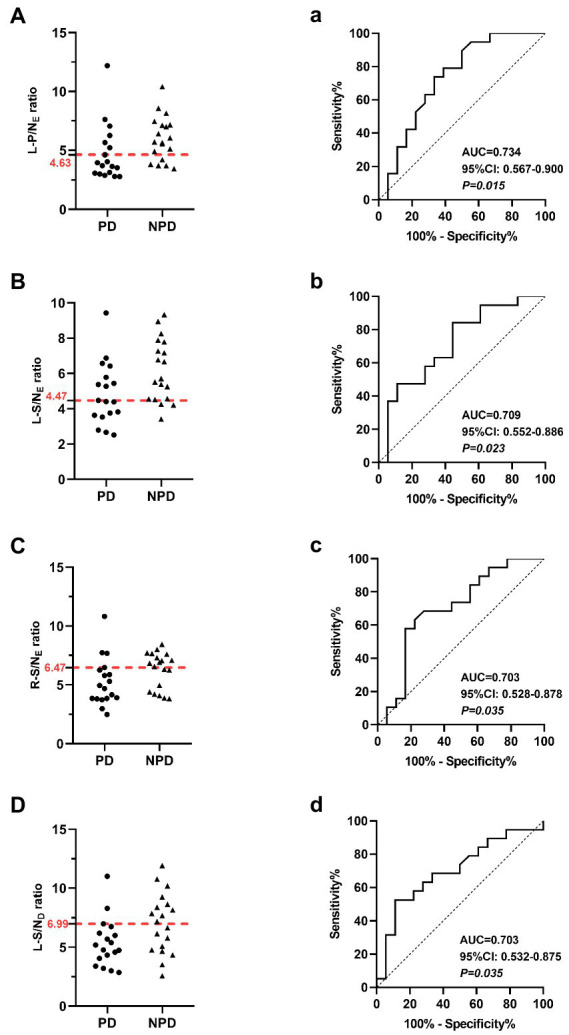
Scatter diagrams show salivary gland MIBG scintigraphy results in the differentiation of PD and NPD. **(A)** The left parotid gland uptake ratio in early images. **(B)** The left submandibular gland uptake ratio in early images. **(C)** The right submandibular gland uptake ratio in early images. **(D)** The left submandibular gland uptake ratio in delayed images. The red dotted line indicates a cutoff value that yields the most appropriate sensitivity and specificity. Receiver Operating Characteristic (ROC) analysis showing sensitivity and specificity of the left parotid gland uptake ratio **(a)** the left submandibular gland uptake ratio **(b)** the right submandibular gland uptake ratio **(c)** in early images, and the left submandibular gland uptake ratio in delayed images **(d)** used to differentiate patients between PD and NPD.

In the early salivary gland MIBG scintigraphy, the left parotid gland had a better diagnostic ability; the AUC value was 0.734 (95% CI: 0.567–0.900, *p* < 0.05), the sensitivity was 66.67% and the specificity was 76.68%. The AUC value with the left submandibular gland was 0.703 in the delayed images, greater than 0.5 (95% CI: 0.532–0.875, *p* < 0.05), and the sensitivity and specificity were 88.89 and 52.63%, respectively. The MIBG uptake ratios for major salivary glands and ROC curves in the discrimination of PD from NPD are shown in Figure 2.

**Figure 3:**
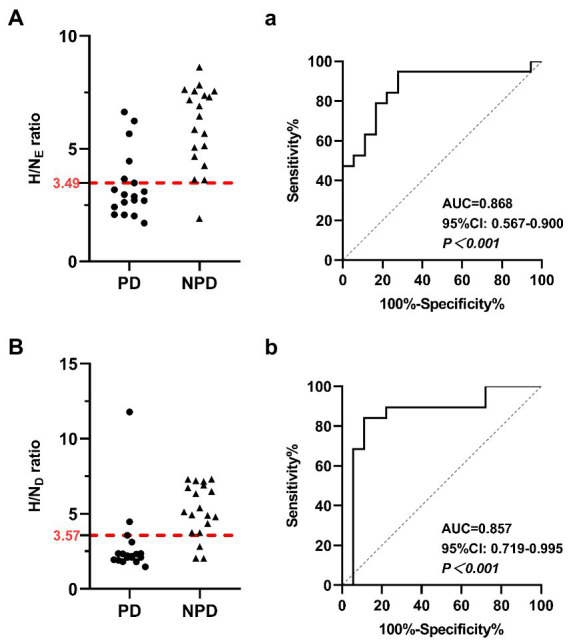
Scatter diagrams show myocardial MIBG scintigraphy results in the differentiation of PD and NPD. **(A)** The heart uptake ratio in early images. **(B)** The heart uptake ratio in delayed images. The red dotted line indicates a cutoff value that yields the most appropriate sensitivity and specificity. Receiver Operating Characteristic (ROC) analysis showing sensitivity and specificity of the heart uptake ratio in early images **(a)** and the delayed images **(b)** used to differentiate patients between PD and NPD.

Similarly, myocardial MIBG scintigraphy exhibited better AUC values for distinguishing PD from NPD in both the early and delayed images. At the OCVs for the myocardial MIBG uptake ratios from early and delayed images, the AUCs were 0.868 (95% CI: 0.567–0.900, *p* < 0.001) and 0.857 (95% CI: 0.719–0.995, *p* < 0.001), the sensitivities were 72.22 and 88.89%, and the specificities were 94.74 and 84.21%, respectively. Figure 3 shows the relevant analysis results.

With the MIBG imaging of salivary glands and myocardium at both periods, we screened out the regions with significant differences in the comparative analysis between the two groups, and performed ROC curve analysis for a diagnosis based on all logical combinations. Among all the multiple indicators of the combination of major salivary glands or the combination of salivary glands and myocardium, we concluded that the best AUC was 0.947 (95% CI: 0.872–1, *p* < 0.001), the sensitivity and specificity were 83.33 and 100% respectively, and the accuracy was 91.67%, which was obtained when we combined all the meaningful indicators in the early and delayed phase imaging. We found that the largest AUC was also obtained when combining indicators other than the early L-P/N, resulting in a sensitivity and specificity of 88.89 and 94.74% (AUC = 0.947, 95% CI: 0.869–1, *p* < 0.001) respectively, and an accuracy of 91.82%. Considering that this approach can provide a more comprehensive diagnosis in practical clinical applications, we selected the best indicators for diagnosis from the early combinations and from the combinations in the delayed period, to use a combination of fewer indicators while achieving relatively higher diagnostic value. By comparing the area under the ROC curve, we found that the combination of data from the major salivary glands did not yield good diagnostic value with an optimal sensitivity and specificity of 50.00 and 94.74% respectively, in early imaging. The accuracy was 72.37% (AUC = 0.731, 95% CI: 0.568–0.894, *p* = 0.0062), for the combination of L-P/N and L-S/N in the early phase. When the L-P/N in major salivary glands and cardiac indicators were combined in early imaging, we obtained the most appropriate sensitivity, specificity and accuracy, which were 77.78, 94.74, and 86.26%, respectively, (AUC = 0.877, 95% CI: 0.763–0.991, *p* < 0.001).

**Figure 4:**
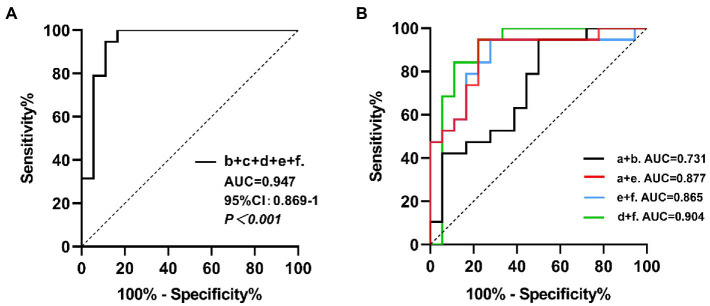
The ROC curves for the combined MIBG scintigraphy uptake ratio. **a**: the left parotid gland uptake ratio in early images; **b**: the left submandibular gland uptake ratio in early images; **c**: the right submandibular gland uptake ratio in early images; **d**: the left submandibular gland uptake ratio in delayed images; **e**: the heart uptake ratio in early images; **f**: the heart uptake ratio in delayed images. **(A)** The b + c + d + e + f combined, AUC = 0.947, 95% CI: 0.869–1, *p* < 0.001. **(B)** The a + b combined, AUC = 0.731, 95% CI: 0.568–0.894, *p* = 0.0062; the a + e combined, AUC = 0.877, 95% CI: 0.763–0.991, *p* < 0.001; the e + f combined, AUC = 0.865, 95% CI: 0.742–0.989, *p* < 0.001; the d + f combined, AUC = 0.904, 95% CI: 0.789–1, *p* < 0.001.

However, in the delayed images, the combination of L-S/N and H/N yielded an AUC of 0.904 (95% CI: 0.789–1, *p* < 0.001), which resulted in a sensitivity of 88.89%, a specificity of 84.21% and an accuracy of 86.55%. Combining only the myocardial MIBG imaging indicators, we obtained the best sensitivity, specificity and accuracy, 72.22, 94.74, and 83.48%, respectively, with an AUC = 0.865 (95% CI: 0.742–0.989, *p* < 0.001). Figure 4 shows the combined diagnostic ROC curves of several optimal combinations.

## 4. Discussion

To our knowledge, this study is the first to use ^131^I-MIBG SPECT/CT imaging for detecting the uptake of sympathetic nerve in major salivary glands and myocardium simultaneously in PD patients. In particular, the ROIs were outlined on the tomographic images. Alhough a few studies have evaluated the sensitivity and specificity of MIBG imaging of the major salivary glands in diagnosing PD, they utilized planar imaging to delineate ROI to quantify the relevant indicators. One study reported that MIBG SPECT had a significantly higher diagnostic performance for PD than planar images (Oh et al., 2015). Furthermore, we collected images at 30 min and 4 h after contrast injection to obtain relevatnt data on the parotid and submandibular glands and myocardium. No study has applied these quantitative indicators together to diagnose PD, including the differential diagnosis of PD and other parkinsonian syndromes. This approach reduces the examination cost and radiation exposure, and also saves time for the patients, especially when compared with previous studies on the diagnosis of PD by combining multiple imaging methods (Sudmeyer et al., 2011; Stathaki et al., 2020).

MIBG is a guanethidine analog that shares the same uptake and storage mechanism as noradrenaline (Spiegel et al., 2005). The ^131^I and ^123^I-labeled MIBG are used in the same principle for myocardial scintigraphy, the latter has relatively good imaging quality, but the short half-life and inconvenient storage of ^123^I-MIBG limit its clinical application. In China, some scholars have made relevant studies on patients with PD, MSA and ET using ^131^I-MIBG myocardial scintigraphy, and proved that ^131^I-MIBG myocardial scintigraphy is helpful in the diagnosis and differential diagnosis of PD (Yang et al., 2017). Our study mainly investigated the complementary role of ^131^I-MIBG major salivary gland and myocardial scintigraphy in the differential diagnosis of parkinsonism, and observed that the combined approach can improve the diagnostic accuracy.

It has been reported that MIBG uptake in the parotid and submandibular glands decreased to different degrees in PD patients during delayed imaging, which is largely normal in MSA patients, PSP patients and healthy subjects (Haqparwar et al., 2017; Soboll et al., 2018; Schubert et al., 2019). This is consistent with our research. In our study, MIBG uptake in the PD group was also significantly lower than that in the NPD group in the early imaging. In particular, the left parotid gland images in the early period and the left submandibular gland images in the delayed period had better specificity and sensitivity in distinguishing the two groups. In view of the small number of patients included in this study and the few previous studies on salivary gland MIBG scintigraphy in PD patients, we cannot determine whether the difference in salivary gland uptake ratio between the left and right sides is related to the side of onset or the side with severe clinical symptoms. However, it is clear that the uptake of salivary glands in early period is related to the MDS-UPDRS III (Supplementary Table 3). In other words, with the aggravation of motor symptoms, the MIBG uptake of salivary glands decreases more significantly.

However, the sensitivity and specificity of major salivary gland MIBG scintigraphy were still relatively low when compared with those of myocardial MIBG scintigraphy. But the combination of diseases common in elderly populations, such as ischemic heart disease and congestive heart failure will also have an impact on the results of myocardial MIBG scintigraphy in PD patients. Therefore, salivary gland MIBG scintigraphy has more important significance as a supplement to myocardial MIBG scintigraphy. The optimal combination of salivary glands was the combination of the left parotid and left submandibular glands in early imaging, with a sensitivity and specificity of 50.00 and 94.74%, respectively, and an accuracy of 72.37%. High specificity has important implications for the clinical exclusion of PD.

The results of salivary gland MIBG scintigraphy can still be used as an important reference, although its sensitivity and specificity were not sufficient to distinguish PD from NPD alone. Because previous studies has confirmed that it is inaccurate to use myocardial MIBG scintigraphy to diagnose PD when patients had heart diseases (Braune, 2001; Jacobson et al., 2010). So, salivary glands MIBG scintigraphy could be used as an important reference index.

In addition to ischemic heart disease, there are several factors that interfere with the use of myocardial MIBG scintigraphy to diagnose PD, such as normal MIBG uptake in some early PD patients. Among the recruited PD patients, 2 PD patients with H&Y stage 1 also had normal myocardial MIBG uptake. Furthermore, myocardial MIBG uptake in some MSA patients was similar to PD patients (Kollensperger et al., 2007; Iwabuchi et al., 2021). In our research results, compared with myocardial MIBG scintigraphy alone, combined scintigraphy improved the sensitivity and specificity of diagnosis, which can reduce the influence of the above factors on the diagnostic value of MIBG imaging to a certain extent.

To facilitate clinical application in the future, we wanted to minimize the number of examinations for patients as much as possible, that is, select only early or delayed image acquisition after contrast agent injection. Therefore in our analysis, we found that combining the left parotid gland and myocardial scintigraphy results in early imaging increased the sensitivity without sacrificing specificity and improved accuracy compared to myocardial scintigraphy alone. When combining the left submandibular gland and myocardial scintigraphy results in delayed imaging, the AUC improved, while the sensitivity and specificity were not reduced. To determine which method is more meaningful, further research is needed. This also provides evidence for major salivary gland MIBG scintigraphy as a supplementary diagnostic basis in Parkinson’s-related disease diagnoses. In this case, imaging of the salivary glands can assist in the diagnosis of PD without repeated examination. Because we injected the contrast agent only once, but could simultaneously collect MIBG images of both the salivary glands and myocardium using SPECT/CT.

However, there are some limitations of our study. First, given that our patients were recruited from a single centre, the sample size was relatively small, and our results might be limited by specific institutional factors. Second, each diagnosis was not pathologically confirmed. Finally, limited local actual conditions, we choose ^131^-MIBG for scintigraphy, while ^123^-MIBG has been widely used for myocardial scintigraphy in previous studies due to its clearer imaging. If the actual conditions permit, using ^123^I-MIBG imaging may make the diagnostic value of MIBG scintigraphy higher.

In addition, artificial intelligence technology includes machine learning such as e classification and regression tree (CART), support vector machines (SVM) and random forest (RF) classifier, plays a certain role in assisting to determine the cutoff values and further diagnosing Parkinson’s syndrome by combing MIBG scintigraphy, and its effectiveness has been well demonstraed in previous studies(Nuvoli et al., 2017, 2020; Iwabuchi et al., 2021). Besides, some studies have applied deep learning to DAT PET and FDG PET scans to analyze multiple regions as well as their correlation, which proved that it was helpful for the early diagnosis of Parkinson’s disease (Wu et al., 2022; Zhao et al., 2022). In future studies, we can also refer to the above methods to simultaneously analyze MIBG uptake in sympathetic distributed organs including salivary glands and heart from SPECT/CT imaging, so as to improve the combined multiorgan MIBG imaging for the differential diagnosis of Parkinson’s disease. At the same time, combined with demographic, clinical data and other indicators to further improve the diagnostic accuracy. In a word, we still need to improve methods to enhance diagnostic performance.

## 5. Conclusion

The sympathetic nerves that innervate the parotid and submandibular glands are damaged in PD patients, mainly manifested by decreased MIBG uptake compared to NPD patients. ^131^I-MIBG salivary gland scintigraphy has certain clinical diagnostic value in the diagnosis and differential diagnosis of PD, especially when PD patients suffer from heart disease and it provides a complementary method. It is important that the combination of ^131^I-MIBG major salivary gland and myocardium scintigraphy improves the accuracy of PD diagnosis and appears to be a feasible method in practical clinical applications.

## Data availability statement

The raw data supporting the conclusions of this article will be made available by the authors, without undue reservation.

## Ethics statement

Written informed consent was obtained from the individual(s) for the publication of any potentially identifiable images or data included in this article.

## Author contributions

QG and LJ designed the research and revised the manuscript. SL drafted the manuscript. SL and LY performed the research, collected and analyzed data. ZW and RH revised the manuscript. SC and CW helped in images acquisition and data analysis. JZ, LX, KP, and AL contributed to data collection and analysis. All authors have read and approved the final version of manuscript for publication.

## Funding

This study was supported by the National Natural Science Foundation of China (Grant number. 81974198); the Fundamental Research Funds for the Central Universities (Grant number. 22120180511); Clinical Technology Innovation Project of Shanghai Shenkang Hospital Development Center (Grant number. SHDC12018X08); and Key Projects Clinical Research Cultivation Project of Tongji Hospital (Grant number. ITJ(ZD)1810).

## Conflict of interest

The authors declare that the research was conducted in the absence of any commercial or financial relationships that could be construed as a potential conflict of interest.

## Publisher’s note

All claims expressed in this article are solely those of the authors and do not necessarily represent those of their affiliated organizations, or those of the publisher, the editors and the reviewers. Any product that may be evaluated in this article, or claim that may be made by its manufacturer, is not guaranteed or endorsed by the publisher.
