# Supplementary material for: High clinical diagnostic accuracy of combined salivary gland and myocardial metaiodobenzylguanidine scintigraphy in the diagnosis of Parkinson’s disease
Source: Front Aging Neurosci. 2023 Jan 11;14:1066331. doi: 10.3389/fnagi.2022.1066331 (PMC9875016; doi:10.3389/fnagi.2022.1066331)
Supplement: Supplementary file 1 [file Table_1.DOCX]

**Supplementary Table 1. Characteristics and the MIBG uptake ratios of PD patients.**

| **Subject Identification** | **Sex**  **(F/M)** | **Age**  **(years)** | **Early ratios** | | | | |  | **Delayed ratios** | | | | |
| --- | --- | --- | --- | --- | --- | --- | --- | --- | --- | --- | --- | --- | --- |
|  |  |  | **L-P/N** | **R-P/N** | **L-S/N** | **L-P/N** | **H/N** |  | **L-P/N** | **R-P/N** | **L-S/N** | **L-P/N** | **H/N** |
| 1 | F | 50 | 3.72 | 5.49 | 4.39 | 3.81 | 2.42 |  | 4.89 | 6.60 | 5.69 | 4.07 | 1.82 |
| 2 | M | 59 | 2.90 | 3.42 | 2.79 | 4.16 | 2.08 |  | 4.90 | 7.22 | 4.74 | 4.99 | 1.91 |
| 3 | M | 55 | 3.97 | 4.38 | 3.82 | 4.94 | 3.66 |  | 4.72 | 5.03 | 4.58 | 4.39 | 2.11 |
| 4 | M | 54 | 5.22 | 5.55 | 6.42 | 6.25 | 3.19 |  | 4.98 | 4.79 | 6.01 | 6.51 | 2.32 |
| 5 | M | 52 | 3.54 | 5.62 | 4.47 | 3.84 | 2.89 |  | 4.45 | 5.48 | 3.39 | 3.82 | 2.20 |
| 6 | F | 54 | 3.08 | 4.15 | 5.27 | 4.68 | 3.10 |  | 4.43 | 4.34 | 4.33 | 4.92 | 2.11 |
| 7 | M | 55 | 7.62 | 7.11 | 6.57 | 7.68 | 2.97 |  | 8.36 | 8.21 | 8.31 | 8.78 | 2.36 |
| 8 | M | 75 | 2.81 | 3.00 | 3.63 | 3.73 | 2.02 |  | 4.21 | 5.81 | 5.39 | 5.90 | 2.36 |
| 9 | F | 43 | 5.65 | 6.83 | 6.87 | 5.79 | 6.24 |  | 8.46 | 11.57 | 11.01 | 9.72 | 11.78 |
| 10 | M | 63 | 6.25 | 9.23 | 5.37 | 7.73 | 4.45 |  | 4.42 | 6.63 | 3.00 | 4.10 | 2.18 |
| 11 | F | 42 | 4.06 | 6.99 | 5.44 | 5.29 | 6.64 |  | 4.68 | 4.97 | 4.06 | 3.95 | 3.57 |
| 12 | M | 71 | 12.20 | 10.80 | 4.40 | 5.87 | 2.70 |  | 8.40 | 7.37 | 3.20 | 3.80 | 2.09 |
| 13 | F | 43 | 3.66 | 4.62 | 2.51 | 2.48 | 2.72 |  | 7.73 | 8.33 | 6.19 | 7.00 | 3.12 |
| 14 | M | 46 | 2.80 | 2.17 | 2.66 | 2.97 | 2.07 |  | 6.10 | 4.83 | 4.77 | 5.45 | 2.33 |
| 15 | M | 72 | 3.01 | 3.83 | 3.53 | 3.84 | 2.63 |  | 6.48 | 7.82 | 5.19 | 5.15 | 1.80 |
| 16 | M | 67 | 4.63 | 5.49 | 5.78 | 6.47 | 1.70 |  | 6.13 | 7.55 | 6.99 | 6.97 | 1.47 |
| 17 | M | 76 | 7.05 | 8.19 | 9.43 | 10.82 | 5.67 |  | 11.30 | 9.26 | 6.75 | 8.92 | 4.47 |
| 18 | F | 60 | 3.14 | 3.46 | 3.75 | 3.90 | 3.49 |  | 3.52 | 3.67 | 2.85 | 3.99 | 1.94 |

MIBG, metaiodobenzylguanidine; PD, Parkinson’s disease. L, left side; R, right side; P/N, the ratio of the parotid glands to the neck subcutaneous tissue; S/N, the ratio of the submandibular glands to the neck subcutaneous tissue; H/N, the ratio of the heart to the neck subcutaneous tissue.

**Supplementary Table 2. Characteristics and the MIBG uptake ratios of non-PD patients.**

| **Subject Identification** | **Final Diagnosis** | **Sex**  **(F/M)** | **Age**  **(years)** | **Early ratios** | | | | |  | **Delayed ratios** | | | | |
| --- | --- | --- | --- | --- | --- | --- | --- | --- | --- | --- | --- | --- | --- | --- |
|  |  |  |  | **L-P/N** | **R-P/N** | **L-S/N** | **L-P/N** | **H/N** |  | **L-P/N** | **R-P/N** | **L-S/N** | **L-P/N** | **H/N** |
| 1 | MSA | F | 47 | 3.46 | 4.96 | 5.71 | 4.39 | 5.86 |  | 5.58 | 6.58 | 5.8 | 5.95 | 7.2 |
| 2 | MSA | F | 56 | 3.72 | 4.22 | 3.41 | 4.21 | 4.26 |  | 6.3 | 6.78 | 6.16 | 6.09 | 4.93 |
| 3 | MSA | M | 65 | 7.17 | 9.33 | 4.21 | 6.32 | 7.3 |  | 6.16 | 8.09 | 4.67 | 5.9 | 6.73 |
| 4 | MSA | M | 70 | 7.00 | 6.60 | 5.38 | 3.82 | 3.64 |  | 13.09 | 11.22 | 7.69 | 7.28 | 5.13 |
| 5 | MSA | F | 50 | 6.41 | 8.59 | 7.79 | 7.72 | 8.63 |  | 7.55 | 7.65 | 9.27 | 8.28 | 7.30 |
| 6 | MSA | M | 64 | 7.13 | 7.90 | 7.17 | 6.90 | 3.62 |  | 9.06 | 8.92 | 8.40 | 7.37 | 2.04 |
| 7 | MSA | F | 59 | 3.73 | 3.89 | 4.52 | 4.95 | 4.66 |  | 4.61 | 4.20 | 4.35 | 4.89 | 2.82 |
| 8 | MSA | F | 72 | 8.15 | 9.47 | 9.34 | 8.45 | 5.14 |  | 9.77 | 11.40 | 10.77 | 9.42 | 3.74 |
| 9 | Unclassified | F | 62 | 8.57 | 7.07 | 8.25 | 7.61 | 5.06 |  | 7.23 | 8.50 | 8.17 | 6.44 | 3.75 |
| 10 | Unclassified | M | 65 | 4.23 | 6.36 | 4.55 | 6.83 | 1.91 |  | 11.44 | 12.84 | 11.92 | 8.41 | 2.03 |
| 11 | Unclassified | F | 67 | 7.45 | 9.66 | 6.67 | 7.66 | 5.69 |  | 10.42 | 13.07 | 10.2 | 9.24 | 5.39 |
| 12 | PSP | F | 78 | 5.65 | 5.94 | 4.57 | 6.56 | 7.63 |  | 5.45 | 4.17 | 5.08 | 4.06 | 4.79 |
| 13 | VaP | M | 75 | 4.91 | 4.31 | 4.25 | 4.08 | 6.45 |  | 3.70 | 4.28 | 2.57 | 4.04 | 4.36 |
| 14 | HS | F | 65 | 10.41 | 8.16 | 8.95 | 8.01 | 7.56 |  | 6.68 | 6.63 | 7.19 | 6.88 | 4.89 |
| 15 | HS | F | 72 | 5.54 | 6.05 | 6.77 | 6.25 | 7.84 |  | 6.29 | 5.77 | 4.77 | 5.38 | 6.90 |
| 16 | HS | F | 49 | 5.70 | 5.59 | 5.25 | 3.88 | 7.57 |  | 4.29 | 5.12 | 3.52 | 4.47 | 6.34 |
| 17 | HS | F | 70 | 6.06 | 7.25 | 7.27 | 7.05 | 7.37 |  | 6.08 | 6.38 | 6.66 | 6.96 | 6.49 |
| 18 | HS | M | 65 | 3.83 | 4.97 | 5.51 | 7.08 | 6.90 |  | 5.64 | 8.43 | 7.87 | 8.79 | 7.29 |
| 19 | HS | M | 64 | 5.12 | 7.04 | 7.89 | 7.30 | 7.17 |  | 7.07 | 7.52 | 8.66 | 7.70 | 7.15 |

MIBG, metaiodobenzylguanidine; PD, Parkinson’s disease; MSA, multiple system atrophy; PSP, progressive supranuclear palsy; VaP, vascular parkinsonism; Unclassified, unclassified parkinsonian syndrome; HS, healthy subjects. L, left side; R, right side; P/N, the ratio of the parotid glands to the neck subcutaneous tissue; S/N, the ratio of the submandibular glands to the neck subcutaneous tissue; H/N, the ratio of the heart to the neck subcutaneous tissue.

**Supplementary Table 3. Correlation between clinical data and the MIBG uptake ratios in PD patients.**

|  | **Disease duration** | |  | **MDS-UPDRS(Ⅲ)** | |
| --- | --- | --- | --- | --- | --- |
|  | ***r value*** | ***P value*** |  | ***r value*** | ***P value*** |
| **L-P/N_E_** | -0.175 | 0.488 |  | -0.616 | 0.007^**^ |
| **R-P/N_E_** | -0.156 | 0.536 |  | -0.583 | 0.011^*^ |
| **L-S/N_E_** | -0.133 | 0.598 |  | -0.393 | 0.107 |
| **R-S/N_E_** | -0.124 | 0.624 |  | -0.529 | 0.024^*^ |
| **H/N_E_** | -0.286 | 0.249 |  | -0.457 | 0.057 |
| **L-P/N_D_** | -0.051 | 0.842 |  | -0.271 | 0.277 |
| **R-P/N_D_** | -0.152 | 0.547 |  | -0.136 | 0.590 |
| **L-S/N_D_** | -0.003 | 0.990 |  | +0.012 | 0.961 |
| **R-S/N_D_** | -0.109 | 0.668 |  | -0.051 | 0.841 |
| **H/N_D_** | -0.277 | 0.265 |  | -0.240 | 0.338 |

Spearman’s correlation was used to calculate the correlation coefficient among variables. MIBG, metaiodobenzylguanidine; PD, Parkinson’s disease. L, left side; R, right side; E, early period; D, delayed period. P/N, the ratio of the parotid glands to the neck subcutaneous tissue; S/N, the ratio of the submandibular glands to the neck subcutaneous tissue; H/N, the ratio of the heart to the neck subcutaneous tissue. ^*^*P*＜0.05，^**^*P*＜0.01
